# Wnt5a Is Associated with Cigarette Smoke-Related Lung Carcinogenesis via Protein Kinase C

**DOI:** 10.1371/journal.pone.0053012

**Published:** 2013-01-21

**Authors:** Young Mi Whang, Ukhyun Jo, Jae Sook Sung, Hyun Jung Ju, Hyun Kyung Kim, Kyong Hwa Park, Jong Won Lee, In Song Koh, Yeul Hong Kim

**Affiliations:** 1 Department of Oncology/Hematology and Brain Korea 21 Project for Biomedical Science, Korea University Anam Hospital, Korea University College of Medicine, Seoul, Korea; 2 Department of Physiology, College of Medicine, Hanyang University, Seoul, Republic of Korea; Univesity of Texas Southwestern Medical Center at Dallas, United States of America

## Abstract

Wnt5a is overexpressed during the progression of human non-small cell lung cancer. However, the roles of Wnt5a during smoking-related lung carcinogenesis have not been clearly elucidated. We investigated the associations between Wnt5a and the early development of cigarette smoke related lung cancer using human bronchial epithelial (HBE) cells (NHBE, BEAS-2B, 1799, 1198 and 1170I) at different malignant stages established by exposure to cigarette smoke condensate (CSC). Abnormal up-regulation of Wnt5a mRNA and proteins was detected in CSC-exposed transformed 1198 and tumorigenic 1170I cells as compared with other non-CSC exposed HBE cells. Tumor tissues obtained from smokers showed higher Wnt5a expressions than matched normal tissues. In non-CSC exposed 1799 cells, treatment of recombinant Wnt5a caused the activations of PKC and Akt, and the blockage of Wnt5a and PKC significantly decreased the viabilities of CSC-transformed 1198 cells expressing high levels of Wnt5a. This reduced cell survival rate was associated with increased apoptosis via the down-regulation of Bcl2 and the induction of cleaved poly ADP-ribose polymerase. Moreover, CSC-treated 1799 cells showed induction of Wnt5a expression and enhanced colony-forming capacity. The CSC-induced colony forming efficiency was suppressed by the co-incubation with a PKC inhibitor. In conclusion, these results suggest that cigarette smoke induces Wnt5a-coupled PKC activity during lung carcinogenesis, which causes Akt activity and anti-apoptosis in lung cancer. Therefore, current study provides novel clues for the crucial role of Wnt5a in the smoking-related lung carcinogenesis.

## Introduction

Lung cancer remains a leading cause of cancer-related death worldwide and cigarette smoking is the most significant risk factor in the development of lung cancer [1], [2]. Cigarette smoke contains a complex mixture of carcinogens, co-carcinogens, mutagens and tumor promoters [3]. It is known that components of cigarette smoke can activate a variety of cellular signaling pathways, increasing anti-apoptosis and cell proliferation [4]. Over the last few decades, considerable efforts for the outcome of lung cancer have been made to clarify the biological mechanisms of smoking-induced lung carcinogenesis [1], [5], [6].

Among the reported molecular signaling pathways associated with lung cancer, Wnt gene family is known to participate in lung tumorigenesis [7], [8]. Wnt5a, a member of the Wnt family, is frequently up-regulated in human lung, breast and prostate cancers. The elevated Wn5a expression is indeed associated with a poor prognosis in non-small cell lung cancer (NSCLC), especially in squamous cell carcinomas [9] ^.^ Moreover, Wnt5a plays an important role in normal lung morphogenesis and in the differentiation of pulmonary specific cell types [10] ^.^ Accordingly, these findings suggest that Wnt5a participates in early lung carcinogenesis. However, it remains unclear whether Wnt5a contributes to the development of smoking-related lung cancer.

Protein kinase C (PKC) is a family of genes that contains eleven distinct lipid-regulated protein-serine/threonine kinases, which have different roles in the regulation of cellular signals depending on specific isoforms, cell types, and/or stimuli [11]. Several studies have shown that blockage of PKC activation causes apoptotic cell death in lung cells, suggesting that modulation of PKC may have therapeutic potential in human lung cancer [12], [13]. In addition, carcinogens in cigarette smoke can activate PKC signaling as a signaling mediator to regulate cell growth in lung cancer [14]. Furthermore, Wnt5a can induce activation of PKC via a noncanonical Wnt/Ca^2+^ pathway [15]. Therefore, it is possible that Wnt5a can modulate the PKC signaling pathway in lung epithelial cells exposed to cigarette smoke, however, the relationship between Wnt5a and PKC in lung carcinogenesis remains uncertain.

In this study, we sought to determine whether Wnt5a plays a crucial role in smoking-induced lung carcinogenesis, and to identify the molecular pathway involved. To define the hypothesis, we used human bronchial epithelial (HBE) cell lines at different malignant stages established by exposing them to cigarette smoke condensate (CSC) [16], [17]. We found that Wnt5a expression was increased in smoking-related pre-malignant transformed HBE cells and clinical tissue samples, and PKC was involved in Wnt5a-mediated early development of lung cancer.

## Materials and Methods

### Subjects and reagents

Normal human bronchial epithelial (NHBE) cells (Cambrex Bio Science, Walkersville, MD) were purchased. Normal immortalized (BEAS-2B and 1799), transformed (1198) and tumorigenic (1170-I) HBE cells were obtained as kind gifts from Dr. Curtis Harris (National Institutes of Health, Bethesda, MD) [18] and Dr. Andres Klein-Szanto (Fox Chase Cancer Center, Philadelphia, PA) [16]. NHBE cells were grown in bovine epithelial growth media with SingleQuots supplements and growth factors (bovine pituitary extract, hydrocortisone, human epithelial growth factor, epinephrine, insulin, triiodothyronine, transferrin, gentamicin, amphotericin-B, retinoic acid, and bovine serum albumin-fatty acid free). The BEAS-2B and 1799 cells were grown in keratinocyte serum-free media (Gibco BRL, Eggstein, Germany) supplemented with EGF and pituitary extract. The 1198 and 1170-I cells were cultured in the same medium used in BEAS-2B cells, except that 3% FBS was added to the medium. The five HBE cell characteristics are summarized in Table 1. A549 cell line (American Type Culture Collection, Manassas, VA) was used as a positive control of malignant stage.

**Table 1:** Characteristics of Five Human Brochial Epithelial Cell Lines.

	NHBE	BEAS-2B	1799	1198	1170-I
Histological stage^a^	Normal	Immortalized	Immortalized	Transformed	Tumorigenic
Xenograft passage^a^	**−**	**−**	**+**	**+**	**+**
CSC exposure^a^	**−**	**−**	**−**	**+**	**+**
Colony forming efficiency^b^	low	low	low	high	high
Tumorigenicity^b^	**−**	**−**	**−**	**−**	**+**
TP53 mutation^a^	**−**	**−**	**−**	**−**	**−**
K-ras mutation^a^	**−**	**−**	**−**	**−**	**−**

Abbreviations: NHBE: normal human bronchial epithelial, CSC: cigarette smoke condensate.

a Lacroix et al. [17].

b Klein-Szanto et al. [16].

Lung tumors and matched normal lung tissue samples were obtained from 12 lung cancer patients (Korea Lung Tissue Bank of Korea University Guro Hospital, Seoul, Korea). The study protocol was approved by the Institutional Review Board of Korea University Hospital and all patients provided written informed consent.

LY294002, RO31-8220 and Gö6976 (Calbiochem-Novabiochem, San Diego, CA) were prepared in dimethyl sulfoxide (DMSO). Nicotine, 4-(methylnitrosamino)-1-(3-pyridyl)-1-butanone (NNK) and 12-O-tetradecanoylphorbol-13-acetate (TPA) (Sigma-Aldrich, St. Louis, MI) were prepared in DMSO or ethanol. Recombinant Wnt5a (R&D Systems Inc., Minneapolis, MN) was reconstituted in sterile phosphate buffered saline (PBS) containing 0.1% bovine serum albumin.

### Reverse transcription-polymerase chain reaction (RT-PCR) analysis

Total RNA was extracted with Trizol reagent (Invitrogen, Carlsbad, CA). Briefly, 1 µg of total RNA was reverse transcribed into cDNA and amplified by PCR. The primers and PCR reaction conditions for Wnt5a have been described previously [19]. Glyceraldehyde 3-phosphate dehydrogenase (GAPDH) was used as a positive amplification control. PCR products were visualized on 2% agarose gels with ethidium bromide. The PCR results were scanned and mean intensity ratio of Wnt5a/GAPDH analyzed using ImageJ software (NIH Image, National Institutes of Health, Bethesda, MD; online at: http://rsb.info.nih.gov/ij/).

### Quantitative real-time PCR analysis

Quantitative real-time PCR assay was performed using iQ SYBR Green Supermix (Bio-Rad, Hercuis, CA) according to the manufacturer's instructions. Briefly, 1 ul of reversely transcribed cDNA was used for the PCR reactions with the Wnt5a specific forward primers (CAA CTG GCA GGA CTT TCT CA) and reverse primers (CAT TCT TTG ATG CCT GTC TTC G). The PCR reaction was carried out as follows: initial denaturation at 95°C for 5 min followed by: 40 cycles of denaturation at 95°C for 30 s; annealing at 58°C for 30 s; and extension at 72°C for 1 min. The threshold cycle (CT) was analyzed using the PCR apparatus procedure and the relative copy number ratio of Wnt5a (ΔCT) and GAPDH (ΔCT) to determine the mRNA expression levels of Wnt5a. All PCRs were carried out in duplicates.

### Small interfering RNA (siRNA) transfection

The siRNA duplexes for Wnt5a (D-003939-1) were obtained in a siGENOME SMART pool (Dharmacon Research Inc., Lafayette, CO). The siRNA for PKCα and β were purchased (Santa Cruz Biotechnology, Santa Cruz, CA). siRNAs were transfected into the 1799 cell line using Lipofectamine 2000 (Life Technologies, Carlsbad, CA) for 48 h. Gene knockdown was verified by Western blotting for Wnt5a, PKCα and β. Control cells were treated with scrambled duplex.

### Western blot analysis

Cells were lysed and separated by sodium dodecyl sulfate (SDS)-polyacrylamide gel electrophoresis, and resolved proteins were transferred onto polyvinylidene fluoride (PVDF) membranes. Membranes were then blocked and incubated with specific monoclonal antibodies; Wnt5a (R&D Systems, INC, Minneapolis, MN). Isoforms of phospho-PKC (phospho-PKC antibody sampler kit #9921, cell Signaling, Danvers, MA), phospho-PKC α/βII, phospho-PKC (pan), phospho-Akt, Akt and poly ADP-ribose polymerase (PARP; Cell Signaling, Danvers, MA), Bax and Bcl-2 (Santa Cruz Biotechnology), and β-actin (Sigma Chemicals, St Louis, MO).

### Cell viability analysis

The cell viabilities were determined using trypan blue dye exclusion assay. Proportions of dead cells were determined using a hemocytometer.

### Apoptosis assay

Apoptosis was assessed by a flow cytometry-based terminal deoxyribonucleotide transferase-mediated nick-end labeling (TUNEL) assay using an APO-bromodeoxyuridine (APO-BrdUrd) staining kit (Phoenix Flow Systems, San Diego, CA) according to the manufacturer's instructions.

### Preparation of cigarette smoke condensate (CSC)

CSC was prepared as previous described. Briefly, one cigarette (Research Grade Cigarette, University of Kentucky) was combusted with a Variable Speed Pump (Fisher Scientific, Pittsburgh, PA). The smoke was bubbled through 25 ml keratinocyte-serum free media. The resulting suspension was filtered by a 0.22-µm pore filter. This solution was considered to be 100% CSC and diluted with keratinocyte serum-free media fresh before experiments.

### Colony formation assay

After the treatment of 50% CSC every 48 h for 8 days, the survived cells were further incubated for 2 weeks and used for selecting the cells expressing high or low levels of Wnt5a, and then were measured the levels of Wnt5a expression in the cells. After confirming the expression status of Wnt5a, the cells (1×10^3^) with/without the treatment of PKC inhibitor (2.5 µM Gö6o76) were plated on 6-well plates and cultured for 2 weeks. The cell images of colony formation were taken under the gel doc image analyzer (Bio-Rad, Hercuis, CA). The number and size of the stained colony was measured using ImageJ software (NIH Image, National Institutes of Health, Bethesda, MD).

### Statistical analysis

All statistical analysis was conducted by the Student's *t* test. Analyses were carried out using SPSS (ver. 10.1; SPSS Inc., Chicago, IL, USA). A *P* value of <0.05 was considered significant.

## Results

### Wnt5a was overexpressed in smoking-related HBE cells and lung cancer tissues

**Figure 1:**
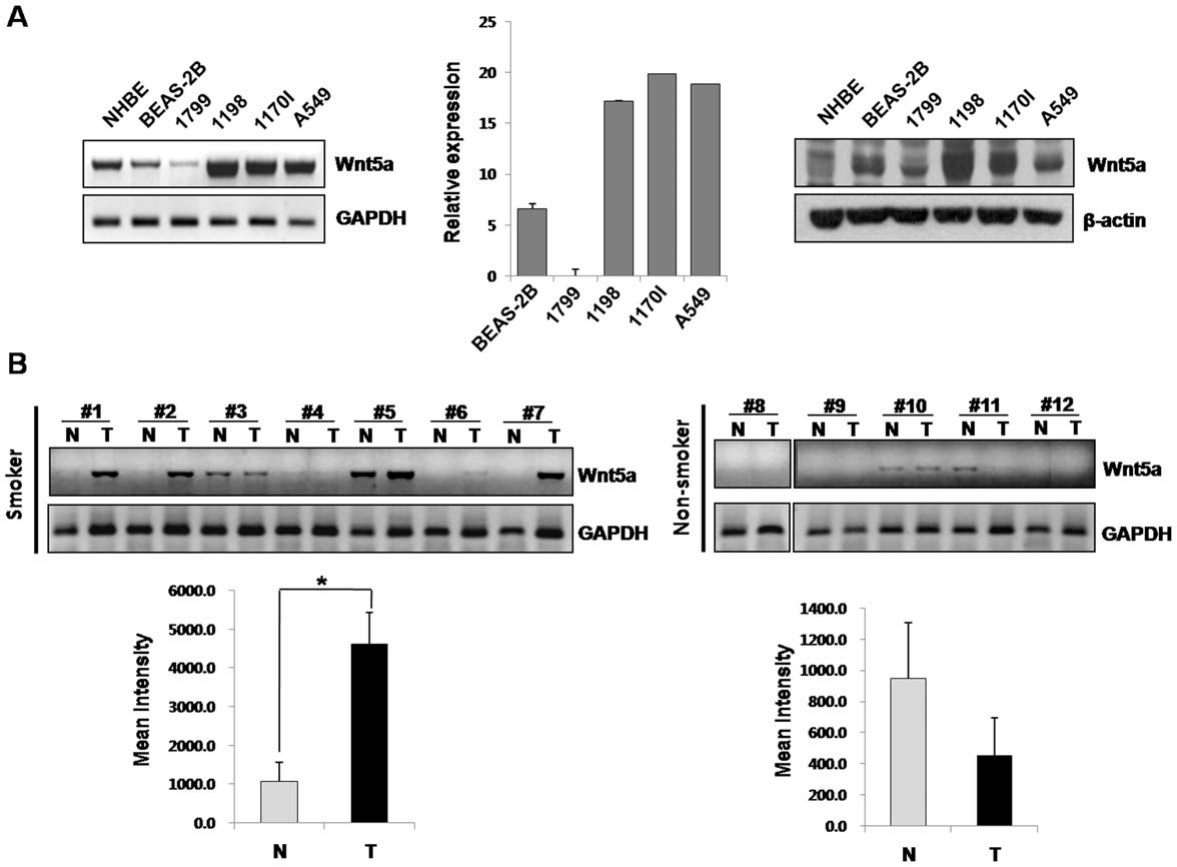
Expression of Wnt5a gene in HBE cells and normal matched lung tissues . (A) mRNA and protein levels of Wnt5a expression were evaluated by RT-PCR (left), quantitative RT-PCR (middle) and Western blot analysis (right) in lung carcinogenesis in five HBE cells (NHBE, BEAS-2B, 1179, 1198 and 1170I) with different cell phenotype by exposure to cigarette smoke condensates. (B) mRNA levels of Wnt5a in 12 normal matched lung tumor tissues (7 smokers and 5 non-smokers) by RT-PCR. N: normal tissue, T: tumor tissue. Quantification of data means the average intensity of Wnt5a bands normalized by GAPDH. Bars denote standard error. Significant differences (*: p<0.03) from normal tissues as determined by a Student's t test.

To investigate whether Wnt5a has a potential role during the early stage of lung cancer development by cigarette smoking, we first profiled Wnt5a mRNA and protein expressions in five HBE cells (NHBE, BEAS-2B, 1179, 1198 and 1170I) at different malignant stages established by exposing them to CSC (Table 1) [16], [17]. We found that the mRNA (Fig. 1A, left and middle panel) and protein expressions of Wnt5a (Fig. 1A, right panel) were increased in CSC-exposed 1198 and 1170I cells versus non-CSC exposed NHBE, BEAS-2B and 1799 cells. A549 lung cancer cells were used as a positive control. We then evaluated the association between Wnt5a expression and smoking exposure using 12 lung cancer samples and matching normal tissues (7 from smokers and 5 from non-smokers) (Figs. 1B). Wnt5a mRNA expression was significantly elevated in 5 of the 7 tumor samples from smokers versus matched normal tissues (*p*<0.05). Interestingly, the tumor samples were obtained from lung cancer patients with squamous cell carcinoma except the #5 sample. However, the statistical association between Wnt5a expression and tumor histology could not be found in the limited sample size. In contrast, no differences in Wnt5a mRNA expressions were found between the tumor and normal tissues of non-smokers. These results imply that Wnt5a is involved in lung carcinogenesis induced by cigarette smoking.

### Wnt5a induced the activations of PKC and Akt in HBE cells

**Figure 2:**
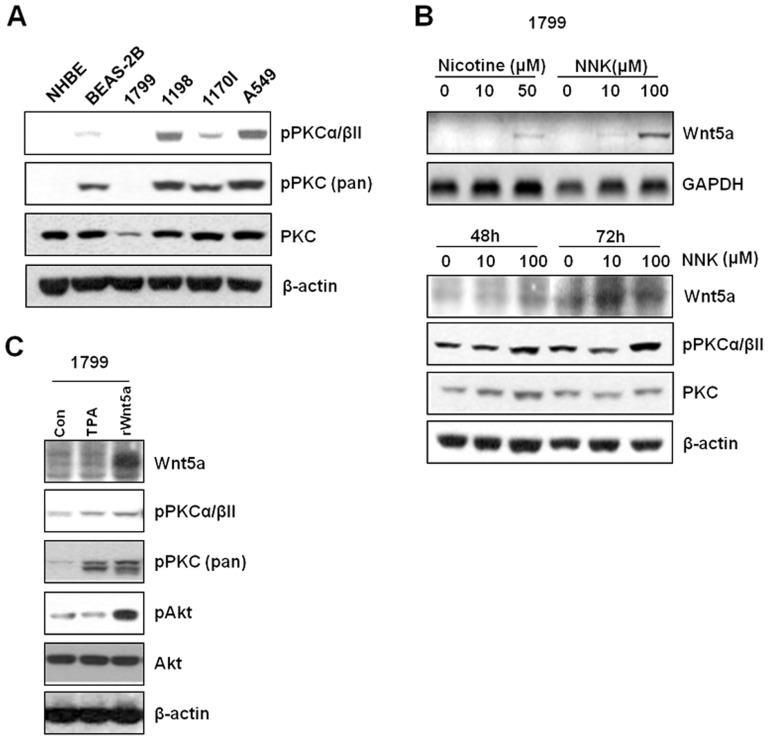
Wnt5a causes activation of PKC and AKT signaling. (A) The phosphorylation status of PKC α/βII and PKC-pan in 5 HBE cells by Western blot analysis. (B) Treatment with cigarette smoking carcinogens, 50 µM nicotin and 100 µM NNK, increases the levels of Wnt5a mRNA expression in 1179 cells. The phosphorylation status of PKC α/βII and PKC-pan was analyzed by Western blot analysis after treatment with 100 µM NNK for 48∼72 h. (C) 1799 cells were treated with recombinant Wnt5a. TPA was used as a positive control to stimulate PKC. β-actin was used as a loading control.

PKC is a molecular mediator regulated by cigarette smoke carcinogens, and a downstream signal transducer in the Wnt5a signaling pathway [15]. To investigate a possible association between PKC and Wnt5a-mediated lung carcinogenesis induced by cigarette smoking, we explored whether activations of PKC isoforms by examining their phosphorylation status in the 5 HBE cells. PKC α/βII and PKC-pan were phosphorylated in response to artificial recombinant Wnt5a (rWnt5a) ligand in the 5 HBE cells but phosphorylation of other isoforms of PKC were not changed (data not shown). In addition, consistent with Wnt5a expression profiles, PKC phosphorylation was found to be increased in CSC- transformed 1198, and tumorigenic 1170I cells as compared with non-CSC exposed HBE cells (Fig. 2A). These results led us to speculate that Wnt5a expression induced by CSC may promote PKC activation and modulate malignant transition from a normal to pre-malignant state. To test this hypothesis, we treated non-CSC exposed 1799 cells that express Wnt5a at low levels with cigarette smoke carcinogens to induce Wnt5a expression. We selected 1799 cells because 1799 cells resemble the characteristics of 1198 cells (e.g. xenograft passages and mutant types of genes) but differ from the 1198 cells only in CSC exposure. Accordingly, we reasoned that 1799 cells provide a better approach than immortalized BEAS2B cells. As a result, treatment with nicotine or NNK increased the expression of Wnt5a mRNA, and this expression was found to be paralleled by PKC α/βII phosphorylation after NNK treatment (Fig. 2B). These results were confirmed by stimulation using rWnt5a into 1179 cells. The exogenously treated Wnt5a induced the phosphorylation of PKC α/βII, PKC-pan and Akt which is a downstream modulator of PKC (Fig. 2C). These results suggest that the activations of PKC and Akt are associated with Wnt5a-mediated pre-malignant transformation.

### The inhibitions of Wnt5a-mediated PKC and Akt signaling decreased the viability of CSC-transformed HBE cells

**Figure 3:**
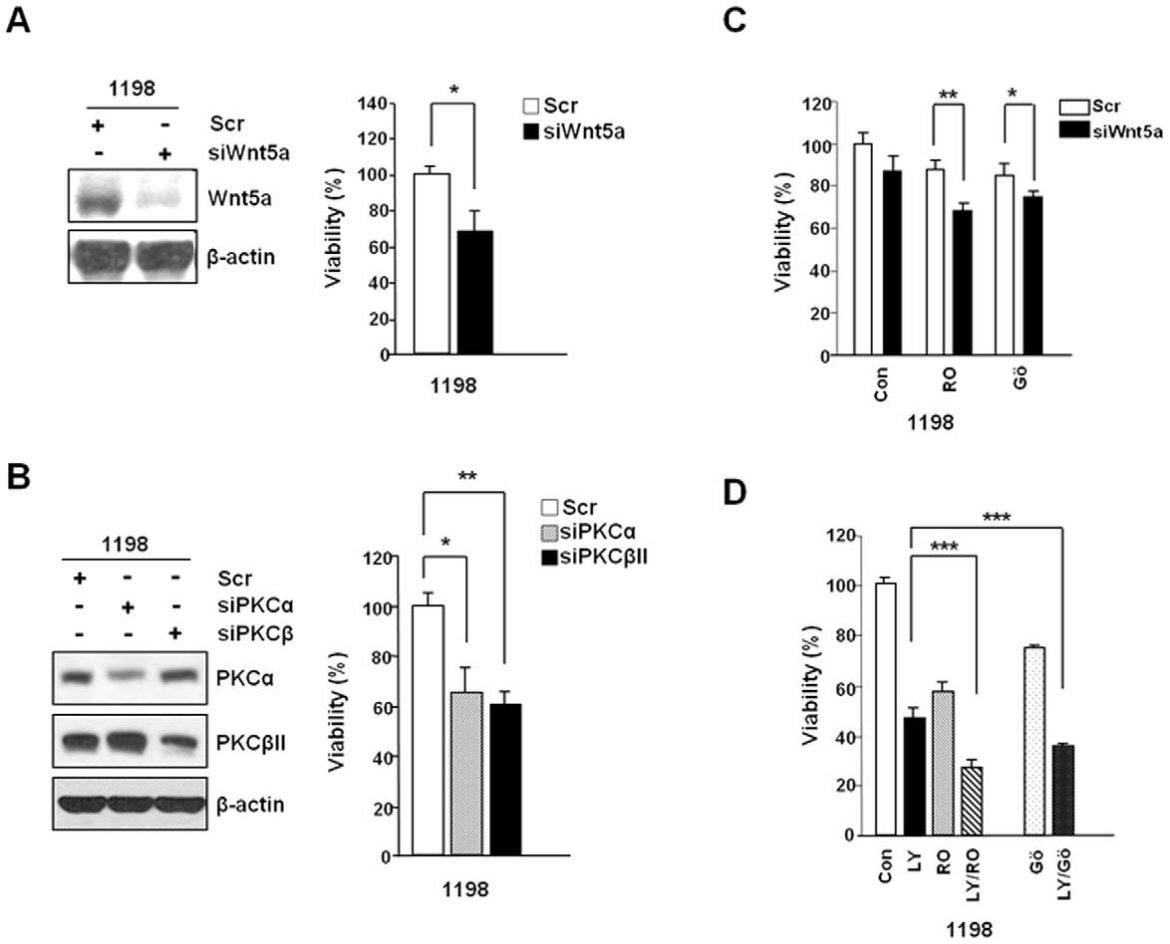
Inhibition of Wnt5a-mediated signaling in pre-malignant HBE cells. (A) 1198 cells were transfected with scrambled siRNA (Scr) and siWnt5a for 48 h. Cell viability was performed by cell counting. (B) 1198 cells were transfected with scrambled siRNA (Scr), siPKCα and siPKCβII for 48 h. Cell viability was performed by cell counting. (C) siRNA transfected 1198 cells were treated with PKC inhibitors (RO: 2.5 µM Ro-318226 and Gö: 2.5 µM Gö6o76) for 24 h and then were counted under a microscope. (D) 1198 cells were treated with PKC inhibitors alone and combination with PI3K inhibitor (LY: 10 µM LY294002).

To determine whether the activations of Wnt5a-mediated PKC and Akt signaling are responsible for abnormal cell proliferation, we used CSC-transformed 1198 cells, which expressed high levels of Wnt5a, representing the earliest stage of lung cancer. In these cells, blockage of the expression of Wnt5a and PKC using siRNAs of Wnt5a and PKC α/βII effectively inhibited the expressions of Wnt5a and PKC α/βII, whereas treatment with scrambled siRNA did not (Figs. 3A left and 3B left). As shown in Figs. 3A right and 3B right, siWnt5a and siPKC α/βII-mediated knockdown significantly reduced the proportion of viable cells as compared with scrambled siRNA transfected cells. Furthermore, combined inhibition with siWnt5a and two specific PKC inhibitors (RO31-8220 and Gö6976) further reduced cell viability (Fig. 3C). These findings were confirmed by blocking PKC and Akt signaling using the two PKC inhibitors and a PI3K/Akt inhibitor (LY294002) in combination (Fig. 3D). These results suggest that Wnt5a signaling via PKC and Akt plays a crucial role in the proliferation of CSC- transformed lung epithelial cells.

### Blockage of Wnt5a-mediated signaling induced apoptotic cell death in CSC-transformed HBE cells

**Figure 4:**
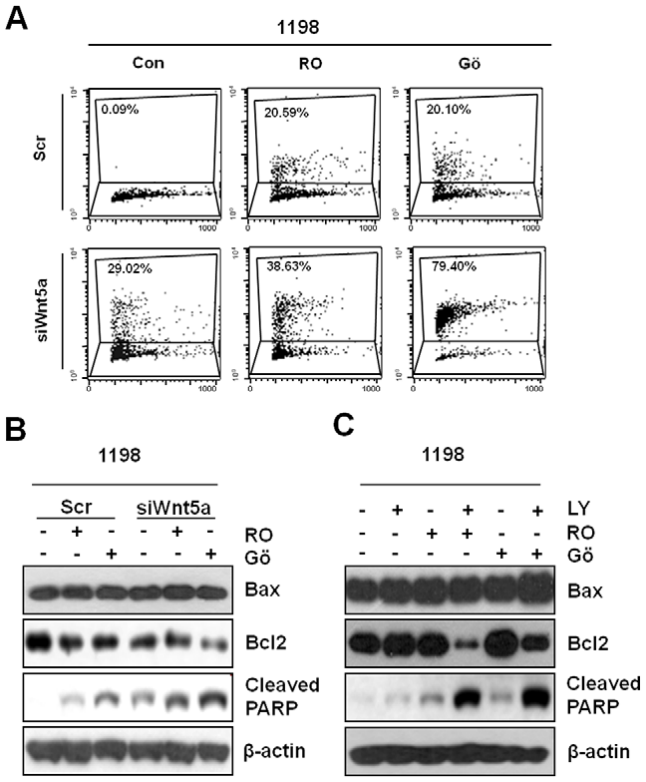
Apoptosis by blocking of siRNA mediated Wnt5a expression and PKC/AKT pathway. (A) 1198 cells were transfected with scrambled siRNA (Scr) or Wnt5a siRNA for 24 h and then treated with PKC inhibitors (RO; 2.5 µM Ro-318226 and Gö; 2.5 µM Gö6o76) ) for 48 h. Apoptosis were determined by the flow cytometric analysis. (B) These cells were analyzed by Western blot analysis of indicated protein expression. (C) 1198 cells were treated with PI3K inhibitor (LY), PKC inhibitors (RO and Gö) or their combination for 72 h and then subjected to Western blot analysis of indicated protein expression. β-actin was used as a loading control.

In order to identify the mechanisms responsible for the reduced cell viability caused by blocking Wnt5a-mediated signaling, we examined apoptotic changes in CSC-transformed 1198 cells by detecting DNA fragmentation using Apo-BrdU. As shown in the Fig. 4A, transfection with siWnt5a significantly increased the proportion of apoptotic 1198 cells, whereas scrambled siRNA had no significant effect on the apoptotic proportion. Furthermore, apoptotic proportions were markedly increased by co-treating the above-mentioned PKC inhibitors as compared with single treatments.

To clarify the molecular features of the apoptotic cell death induced by blocking Wnt5a-mediated signaling, we monitored changes in the levels of the apoptosis related proteins, Bax, Bcl2 and PARP. Knockdown of Wnt5a signaling attenuated Bcl2 protein expression and increased PARP cleavage. In addition, these apoptotic alterations were markedly enhanced by co-administering the two PKC inhibitors (Fig. 4B), and combined treatment with PKC inhibitors and PI3K/Akt inhibitor produced similar effects (Fig. 4C).

### CSC induced the expression of Wnt5a and increased colony forming capacity in non-CSC exposed 1799 cells

**Figure 5:**
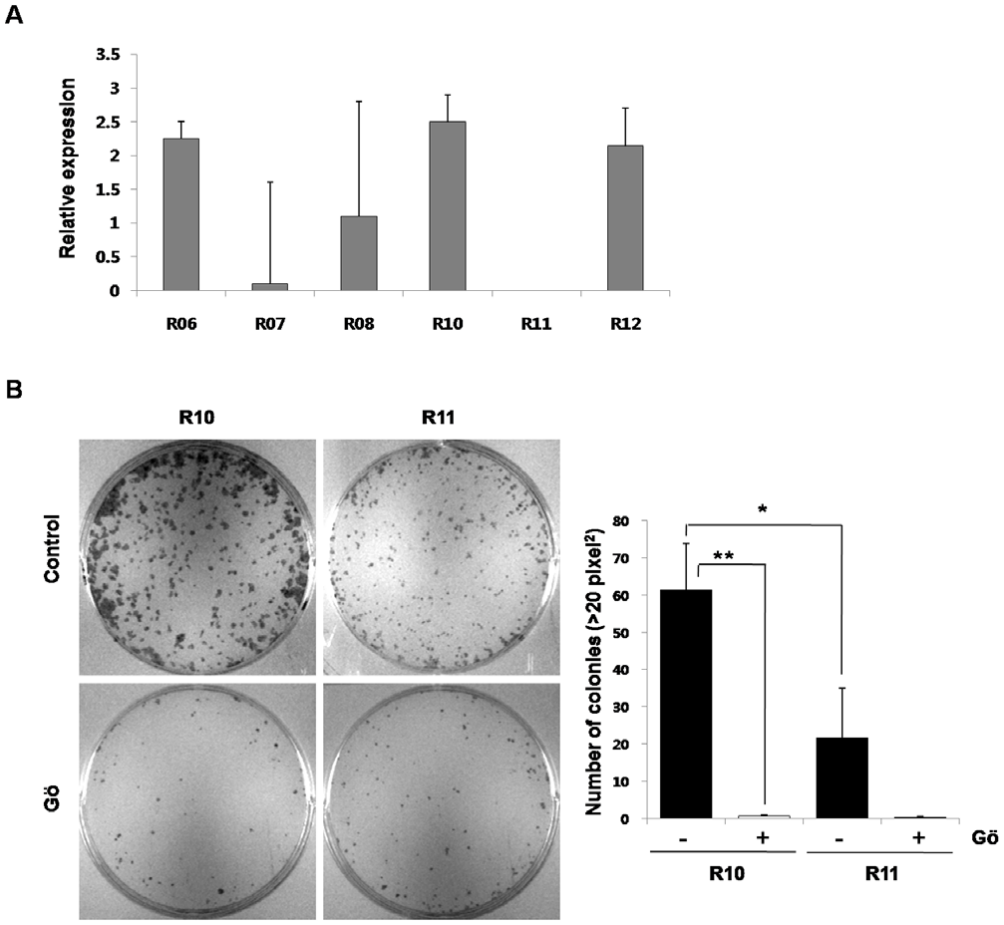
CSC induced the expression of Wnt5a and increased colony forming capacity in non-CSC exposed 1799 cells. (A) 1799 cells treated with 10% CSC for 8 days and further cultured in normal growth medium. CSC-transformed clones of 1799 cells were isolated and used to profile the expression of Wnt5a using quantitative RT-PCR. (B) Selected R10 and R11 clones were examined for the colony-forming efficiency with or without a PKC inhibitor (Gö: 2.5 µM Gö6o76). Colonies were fixed in methanol and stained with 0.1% crystal violet. The data are representative of three experiments. Quantification of data means the average number of colonies (>20 pixel^2^) per dish from the triplicate. Bars denote standard error. Significant differences (*: p<0.05, **: p<0.005) from control as determined by a Student's t test.

Although NNK and nicotine are believed to be the primary tobacco carcinogens, they cannot explain all of cigarette smoke induced carcinogenesis. Thus, we examined the association between Wnt5a and lung cancer in 1799 cells using CSC which contains most of the compounds of cigarette smoke. We cultured 1799 cells with 10% CSC for 8 days to induce temporarily CSC-transformed cells and further cultured 1799 cells in normal growth medium for 2 weeks. Next, we isolated single cell clones with high or low expression of Wnt5a among the CSC-transformed 1799 cells using quantitative RT-PCR (Fig. 5A). We then selected R11 and R10 clones to investigate effects of Wnt5a on colony forming capacity. As shown in the Fig. 5B, R10 clones with high Wnt5a expression showed statistically increased colony number and size compared with that in R11 clones with low Wnt5a expression. Moreover, the colony forming capacity of these clones was inhibited by a PKC inhibitor. Therefore, these results demonstrated that cigarette smoke-related lung carcinogenesis was associated with Wnt5a and PKC.

## Discussion

In the present study, we investigated whether Wnt5a is associated with cigarette smoking induced lung carcinogenesis. Cigarette smoking is the predominant etiologic risk factor of lung cancer development [2]. During previous decades, several molecular factors have been reported to be activated by smoking in the context of lung cancer [20]–[23]. But little is known of the key molecules that act during the earliest stages of lung carcinogenesis induced by cigarette smoking. Therefore, to determine the role of Wnt5a in smoking-induced pre-malignant transformation, we used 5 HBE cells (NHBE, BEAS-2B, 1170, 1198 and 1170I) at different stages of malignancy established by exposing them to CSC [16], [17]. Wnt5a is known to be frequently up-regulated in various human cancers and to act as a tumor promoter [9], [24]. However, the potential role of Wnt5a during pre-malignant transformation in lung cancer remains controversial. Actually, Wnt5a plays an important role in normal lung morphogenesis and in the differentiations of pulmonary specific cell types [10]. Initially, Wnt5a was found to be a non-transforming member of the Wnt family [15], but subsequently, it was found that its acts as a tumor suppressor in human cancers [25], [26]. Nonetheless, Wnt5a seems to act as crucial as an oncogene in lung cancer. In a recent report was shown that the Wnt5a gene is overexpressed in lung cancer, especially in squamous cell carcinoma, and that this overexpression is associated with a poor prognosis in NSCLC patients [9]. Similarly, in the present study, we found Wnt5a was up-regulated in CSC-induced pre-malignant and malignant HBE cells, and in the tumors obtained from smokers. Moreover, the tumors were acquired from lung cancer patients with squamous cell carcinoma, implying possible association between Wnt5a expression and tumor histology like as a previous report [9]. These results suggest that Wnt5a, an adjuvant factor of the morphological differentiation of lung tissues under normal conditions, functions as a tumor promoter when bronchial epithelial cells are injured by smoking carcinogens.

PKC is known to be activated by smoking carcinogens, such as, nicotine and NNK [4], and recently, several researchers have concluded that PKC inhibitors represent a potential strategy for the treatment of human cancers [12], [13]. Actually, a potential association between PKC activation and Wnt5a expression was reported in human melanoma based on the observations that blocking the Wnt5a pathway using specific antibodies inhibited PKC activation, cell motility and invasion [15]. In the present study, we found that the activations of PKC and Akt were involved in the Wnt5a-mediated signaling induced by smoke carcinogens during lung carcinogenesis and in the abnormally high expression of Wnt5a observed. Furthermore, inhibitions of Wnt5a and PKC mediated signaling using siRNAs and small molecule inhibitors reduced the viability of transformed HBE cells. In addition, this reduced viability was found to be associated with apoptosis via Bcl2 down-regulation and PARP cleavage. These results suggest that the modulation of Wnt5a/PKC signaling has therapeutic potential for the prevention of smoking-related lung carcinogenesis.

Taken together, this study shows that Wnt5a-mediated signaling is associated with the pre-malignant transformation of HBE cells during smoking-induced lung carcinogenesis. Abnormally increased Wnt5a expression was detected in pre-malignant and malignant HBE cells, and in the tumor tissues of lung cancer patients with a smoking history, which demonstrated a relationship between Wnt5a signaling and smoking. In addition, Wnt5a induced by known cigarette smoke carcinogens was found to modulate the activations of PKC and Akt to maintain cell viability. Therefore, these findings shed light on the role of Wnt5a during early development of smoking-related lung cancer and suggest potential treatment targets in lung carcinogenesis by cigarette smoke.
